# Identify the Virus-like Models for COVID-19 as Bio-Threats: Combining Phage Display, Spectral Detection and Algorithms Analysis

**DOI:** 10.3390/ijms24043209

**Published:** 2023-02-06

**Authors:** Yuting Wu, Zhiwei Liu, Sihan Mao, Bing Liu, Zhaoyang Tong

**Affiliations:** 1 State Key Laboratory of NBC Protection for Civilian, Beijing 102205, China; 2 School of Chemistry and Chemical Engineering, Nanjing University of Science and Technology, Nanjing 210094, China

**Keywords:** SARS-CoV-2, virus-like models, phage display, identification, spectroscopy, PCA-LDA

## Abstract

The rapid identification and recognition of COVID-19 have been challenging since its outbreak. Multiple methods were developed to realize fast monitoring early to prevent and control the pandemic. In addition, it is difficult and unrealistic to apply the actual virus to study and research because of the highly infectious and pathogenic SARS-CoV-2. In this study, the virus-like models were designed and produced to replace the original virus as bio-threats. Three-dimensional excitation-emission matrix fluorescence and Raman spectroscopy were employed for differentiation and recognition among the produced bio-threats and other viruses, proteins, and bacteria. Combined with PCA and LDA analysis, the identification of the models for SARS-CoV-2 was achieved, reaching a correction of 88.9% and 96.3% after cross-validation, respectively. This idea might provide a possible pattern for detecting and controlling SARS-CoV-2 from the perspective of combining optics and algorithms, which could be applied in the early-warning system against COVID-19 or other bio-threats in the future.

## 1. Introduction

The COVID-19 pandemic has lasted almost three years worldwide since its outbreak in late 2019, affecting all aspects of our lives and activities. And it could be the most severe threat for generations due to its dangerousness and unpredictability [1]. Great efforts have been made to understand and discover SARS-CoV-2, including its viral structure [2,3,4], spread process [5,6,7,8], pathogenesis mechanism [9,10], diagnostic approach [8], variants [11,12,13], and vaccine immunity [13,14,15]. However, there are still significant challenges in the detection of this virus despite all kinds of positive scientific progress have been achieved. After all, an accurate and rapid distinguishment plays an important role in preventing and containing the epidemic. Moreover, early warning and identification of bio-threats is the priority of protection. Considering the operability and timeliness of the technology, detections including sequencing, real-time PCR [16,17,18], loop-mediated isothermal amplification technology (LAMP) [17,19,20,21], droplet digital PCR technology [22], and CRISPR diagnosis [23] have all been developed. Meanwhile, technologies based on serological detection have also performed a significant part in the diagnosis, especially the enzyme-linked immunosorbent assay (ELISA) and lateral flow immunochromatography (LFIA), which have also been often compared to each other to obtain a better sensitivity [24,25,26,27]. However, Methods based on PCR analysis are currently expensive and low-throughput, sometimes resulting in false-negative cases due to procedural or technical problems. High-quality antisera are often required for ELISA techniques, etc. [28]. Most importantly, these methods are destructive to samples. Therefore, it is still a challenge to develop a rapid identification method for SARS-CoV-2.

Spectroscopy is a practical approach for detecting different kinds of viruses [29]; it is vital in bioengineering, natural sciences, environmental monitoring, and medical research. Especially, spectroscopy combined with algorithm analysis can be an effective way to determine the target molecules’ presence. The regular steps for the diagnosis of COVID-19 through spectroscopy could be summarized as follows: (1) collection of specimens from nasopharyngeal or oral swab; (2) preparation of the samples and analysis by spectroscopy such as Raman, infrared, and fluorescence; (3) algorithm applied based on the spectral results; (4) target molecules differentiating from the control samples after statistical analysis. A previous study has proposed a method for detecting COVID-19 by Raman spectroscopy [30]. They analyzed and discriminant against the samples from blood serum by coupling Raman spectroscopy and principal component analysis (PCA) and partial least squares (PLS) with 87% sensitivity and 100% specificity, indicating a possible rapid detection for COVID-19. Apart from that, Sanchez et al. proposed the possibility of detecting spike and nucleocapsid proteins of SARS-CoV-2 using surface-enhanced Raman spectroscopy to replace RT-PCR due to a significant signature of the virus could be obtained [31]. Besides, the team of Barauna reported an onsite, rapid, reagent-free, and nondestructive method for the detection of SARS-CoV-2 by Attenuated total reflectance Fourier transform infrared (ATR-FTIR) spectroscopy and a generic algorithm-linear discriminant analysis (GA-LDA) algorithm, which could lead to a result of 95% for blind sensitivity and 89% for specificity [32]. Except for identifying and detecting SARS-CoV-2, spectroscopy such as three-dimensional excitation-emission matrix fluorescence could be used to classify different proteins [33]. Therefore, spectroscopy combined with machine learning has provided a promising future in detecting and diagnosing viruses such as COVID-19.

The most commonly used algorithms for discriminating are principal component analysis (PCA) and Linear discriminant analysis (LDA). PCA is a dimensionality reduction technology, especially in diverse areas [34]. It can simplify data sets by selecting or transforming them to fewer important variables, also known as principal components, through a linear transformation. This method can extract many original variables with specific correlations, perform feature extraction, and recombine them into a new set of independent and comprehensive indicators to replace the original ones, forming a new minority of variables (components). Using these new variables to replace the original data for subsequent processing, the higher-dimensional space problem can be transformed into a low-dimensional space for analysis [34]. This approach reduces the dimensionality of multivariate data systems and simplifies the statistical features of system variables. PCA has been used in materials and biochemistry areas based on spectra analysis, such as Raman spectra for nanoparticle characterization [35] and Raman spectra for Hepatitis C infection [36], indicating potential in description and screening study. LDA is also a dimensionality reduction technology, which can be concluded as focusing on classifying. When the number of classifications is known, it can be used to determine the category to which an unknown object belongs based on certain observational indicators of the classified objects. The discriminant analytic method needs first to classify the objects, further select some variables that can describe the observation objects more comprehensively, and then establish one or more discrimination functions according to certain discriminant criteria. For a case with an undetermined category, as long as it is substituted into the discrimination function, it can be judged which category it belongs to. The previous study has exhibited that LDA has been used in spectra analysis. Lv et al. proposed to use of LDA to classify freshwater fish species based on near-infrared reflectance spectroscopy [37]. Lin et al. reported a method for identifying osteonecrosis and normal tissue by combining near-infrared spectroscopy and LDA [38]. PCA and LDA both can perform dimensionality reduction on the data [39]. The difference is that LDA is a supervised dimensionality reduction method with instructional values, while PCA is an unsupervised [39]. In addition to dimensionality reduction, LDA can also be used for classification. PCA aims to achieve dimensionality reduction by finding the linear combination with the largest variance of multidimensional data. This method has an obvious influence on simplifying multidimensional data, but it is difficult to achieve effective distinction and distinguishment for similar data belonging to different species. LDA projects high-dimensional data into the best discriminant vector space to maximize the separability of samples in the new space to realize the effective extraction of the classification [39,40]. Therefore, PCA combined with LDA can complement each other and has the advantages of simple procedure and high efficiency. Ren et al. developed a method to recognize asphalt fingerprints based on ATR-FTIR spectroscopy and PCA-LDA analysis [41]. Besides, in the clinical area, PCA-LDA could be employed in diagnosing dental fluorosis with the help of micro-Raman spectroscopy [42]. Also, PCA-LDA could be regarded as a helpful analysis technology in forensic sciences to detect blood strain based on ATR-FTIR [43].

In summary, the combination of spectroscopy and algorithms has been a powerful tool in scientific research, including biochemistry, environment, and forensic studies. However, as for COVID-19, despite all the achievements, there is still limited research on developing a systematic pattern for the early identification of SARS-CoV-2 because the realistic factor, such as the infectivity and pathogenicity of the virus, could not be solved. Moreover, although the distinguishment between SARS-CoV-2 and non-SARS-CoV-2 was realized with an accuracy of 97.4% using complementary DNA and machine learning algorithms [44], the significant role of spectroscopy was neglected. Referring to our previous work [45], the virus-like model based on the nucleocapsid protein of SARS-CoV-2 was synthesized successfully to replace the original virus in some scientific research. Therefore, in this study, spike and nucleocapsid proteins were focused on producing the virus-like models by phage display, which were named Model-S and Model-N, on being substitutes for the bio-threats of COVID-19. After that, three-dimensional excitation-emission matrix fluorescence and Raman spectroscopy were applied to the models and other selected samples for spectral analysis. Followed by the combination of PCA and LDA algorithms, a systematic process to identify and discriminant the virus-like models of COVID-19 was developed. The results from the two types of spectra could be compared. The idea and system may provide a method for actual SARS-CoV-2 detection and help to realize early monitoring for different kinds of bio-threats in the future.

## 2. Results

### 2.1. Preliminary Validation of the Virus-like Model

The Model-N was validated comprehensively in our previous work [45]. In this section, Model-S was verified and proved to be an effective nonpathogenic substitution for SARS-CoV-2 from the insertion of the S gene, the expression of the S protein, the infectivity of the virus, and the affinity analysis with the corresponding antibody.

#### 2.1.1. Molecular Level: The Insertion of the S Gene

**Figure 1:**
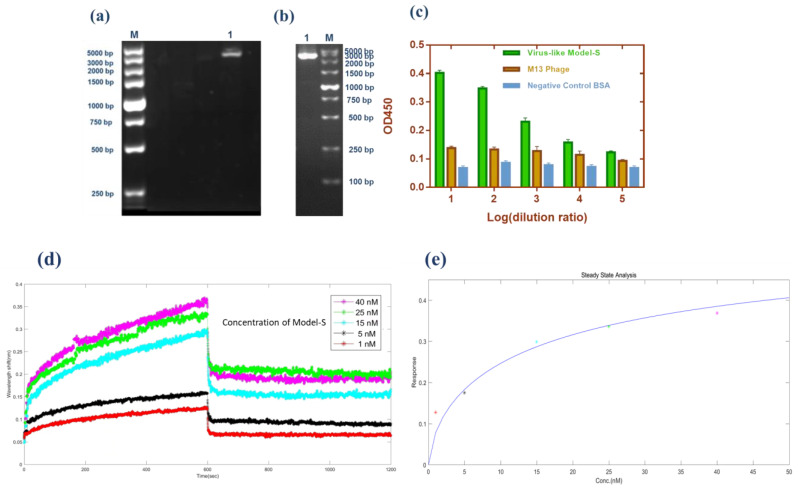
Validation of the Model-S. (**a**) Double digestion (*Sfi*I/*Not*I) of pHB-S. Lane M: Marker; Lane 1: double digestion result. (**b**) PCR amplification of pHB-S. Lane M: Marker; Lane 1: PCR result. (**c**) Sandwich ELISA result from Model-S. Compared with the blank M13 phages and BSA, the results from Model-S were positive when combined with anti-SARS-CoV-2 spike antibodies. (**d**) Affinity analysis: interference shifts over time, reflecting the interference change caused by different concentrations. (**e**) Steady-state analysis: Response over the concentration of Model-S. (**d**,**e**) for the calculation of the affinity constant of Model-S.

As described in the method section, the pHB-S plasmid was reconstructed and prepared for further transformation. Double digestion (*Sfi*I/*Not*I), PCR amplification (Primer-F: 5’-GGCCCAGCCGGCCATGTTTGTTTTTCTTGTTTTATTG-3’; Primer-R: 5’-ATTTGCGGCCGCTTATGTGTAATGTAATTTGACTCC-3’), and sequencing (in Supplementary Information S1.) were completed to prove the correct insertion of the fragment. Figure 1a,b show these two methods’ results, respectively. Obviously, the bands from both methods corresponded to the inserted parts (~3843 bp). Moreover, in brief, the fragments of the S gene were inserted successfully from the molecular level.

#### 2.1.2. Protein Expression: The Display of S Protein

Phage display technology was used to fuse the S protein of SARS-CoV-2 with the p3 protein of the M13 phages. In order to confirm the expression of S protein on the constructed Model-S, the enzyme-linked immunosorbent assay (ELISA) was conducted. In the ELISA experiment, the original M13 phages and BSA were used as the controls. As Figure 1c illustrates, the Model-S showed positive results for the anti-S antibodies compared with the control samples, which may indicate the successful expression of S protein on the Model-S.

#### 2.1.3. Possibility for Being a Substitution: The Infectivity and Affinity Analysis

It is significant to investigate whether the Model-S possess the possibility of infection, and it is the core to evaluate if it can be used as a possible nonpathogenic replacement for SARS-CoV-2. In this study, the titer of the constructed Model-S was measured (see Table 1). According to the plaque assay results, the Model-S titer was 2.42 × 10^9^ pfu/mL. Although, compared with the 8.40 × 10^10^ pfu/mL of the blank M13 phages, the infectivity decreased gently by two orders of magnitude. The produced Model-S remained the strong infectivity as a virus. Also, the affinity analysis was applied between the Model-S and the corresponding anti-SARS-CoV-2 spike protein antibodies. After monitoring the reactions between the antibodies and Model-S from different concentrations (Figure 1d), steady-state analysis was performed, and the relationship between the concentration of Model-S and the response was presented in Figure 1e. The affinity constant was calculated as 4.044 × 10^8^ M^−1^, showing a reasonably well connection between the model and the antibody (The detailed explanation of the affinity analysis was in Supplementary Information S2).

Therefore, it could be concluded that the nonpathogenic virus-like Model-S was successfully constructed based on the spike protein of SARS-CoV-2. Furthermore, all the results from the validation experiments have proved their potential to be a replacement for the further study of COVID-19. Combined with the Model-N proposed in our previous work, these two models were prepared and regarded as the bio-threats in the following analysis.

### 2.2. Three-Dimensional Fluorescence Spectroscopy Analysis and Classification

3DFS for the nine samples were shown in Supplementary Information S3. The fluorescence spectra, contour map, excitation spectra, and emission spectra were obtained from data preprocessing. Kaiser-Meyer-Olkin (KMO) statistics and the Bartlett test of sphericity were used to determine the suitability for PCA analysis. The KMO value is supposed to be between 0 and 1, and the larger it is, the more suitable for conducting the PCA algorithm. Moreover, Bartlett’s test of sphericity hypothesizes that the coeffective correlation matrix is a unit. In this analysis, the KMO was tested as 0.724, and the significance value for Bartlett was 0.000, meaning the hypothesis was rejected. Moreover, there was a relation between the variables, indicating it was suitable for PCA analysis. Table 2 shows the variance contribution for every new principal component and the cumulative contribution percentage.

**Figure 2:**
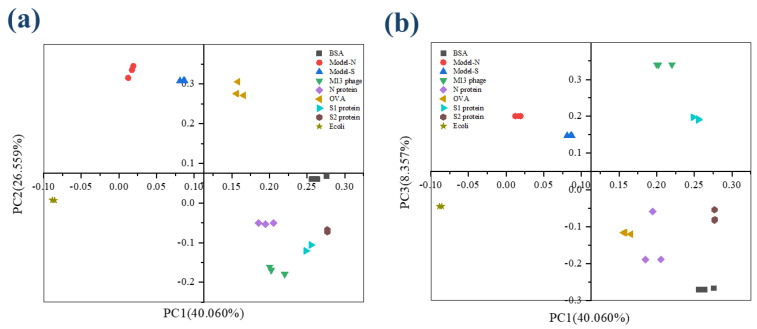
The score plot for the nine samples from 3DFS after PCA analysis. (**a**) Coordination PC1−PC2; (**b**) Coordination PC1−PC3.

In PCA analysis, the cumulative contribution percentage can be accepted when reaching 60% and regarded as a good dimension reduction when reaching 80%. In this study, the cumulative contribution for the first four PCs was 81.949%, with approximately 19.000% of information lost. Therefore, the four components, PC1, PC2, PC3, and PC4, could explain the original variables well. According to the component matrix obtained from PCA reduction, the score scatters diagram for the nine samples were concluded and plotted under the coordinate PC1-PC2 and PC1-PC3 (Figure 2a,b). It is obvious that the nine samples, including the bio-threats, were separated successfully.

**Figure 3:**
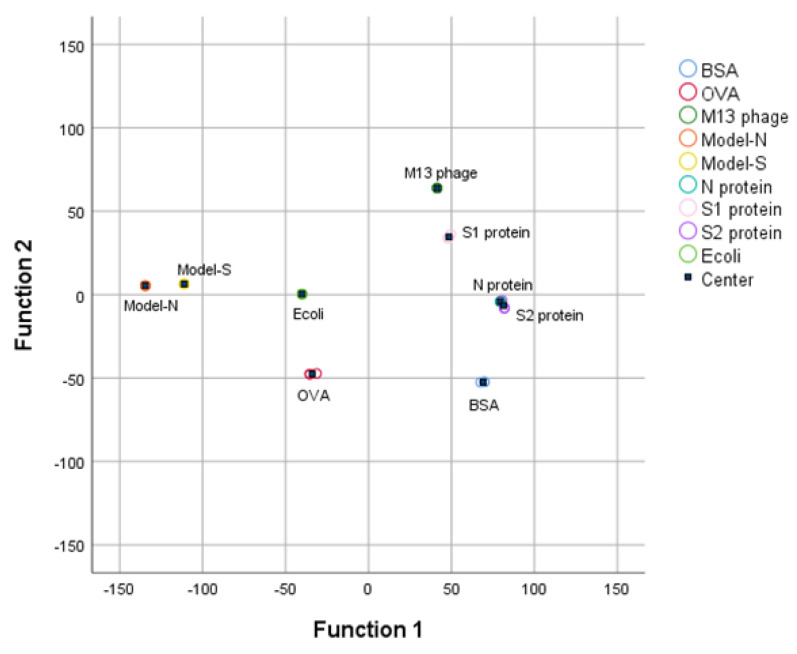
3DFS Group center plot for the nine samples after LDA analysis under the coordination Function 1 and Function 2. Bio−threats Model-S and Model-N can be separated and distinguished from the other samples.

Then, the first four PCs were studied and added with group variables as a training sample set, and the data was processed by linear discriminant analysis (LDA). The first two linear discriminant functions were obtained and validated, as Table 3 shows, and the group center plot was drawn with a coordinate Function 1–2 based on LDA analysis (Figure 3). It can be summarized that the nine samples were separated, and the distribution of the samples was concentrated generally, which might show a possibility for the identification and distinguishment of the virus-like models from the other substances.

In order to confirm the stability and reliability of the functions summarized from LDA analysis, the results were cross−verified and shown in Table 4. 88.9% of the samples were classified correctly. Model−S and Model−N were distinguished from the other samples. Therefore, the virus-like models of SARS−CoV−2 were successfully identified and discriminated by the combination of 3DFS and PCA−LDA methods.

### 2.3. Raman Spectroscopy and Classification

**Figure 4:**
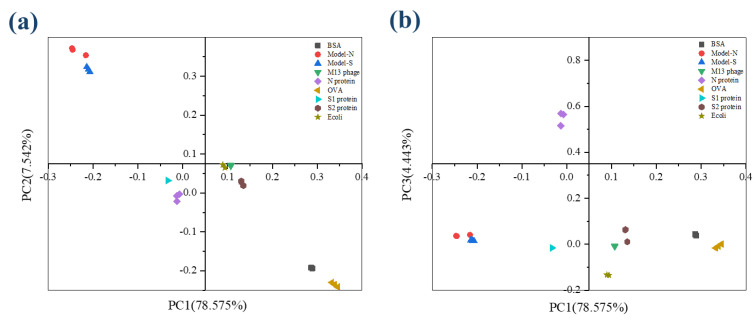
The scatter plot describes the scores for the nine samples from RS after PCA analysis. (**a**) Coordination PC1−PC2; (**b**) Coordination PC1−PC3.

The figures of Raman spectroscopy for the nine samples were shown in Supplementary Information S4. The KMO (Kaiser-Meyer-Olkin) statistic was estimated at 0.895, and the significance from the Bartlett test of sphericity was 0.000, representing that the data from Raman spectra was effective and suitable for PCA analysis. The variance contribution statistics are displayed in Table 5. The variance contributions of the first three principal components were 78.585%, 7.542%, and 4.443%, leading to a cumulative result of 90.559% with less than 10.000% information lost. According to the description above, over 80% may represent an effective and reliable explanation for the original data. The first three components were selected and analyzed in further study to maintain more information from the original data. Similar to the analysis process of 3DFS, the scatter diagram for the scores of PCs under the coordinates PC1-PC2 and PC1-PC3 (shown in Figure 4). The nine samples could be separated and distinguished from each other.

**Figure 5:**
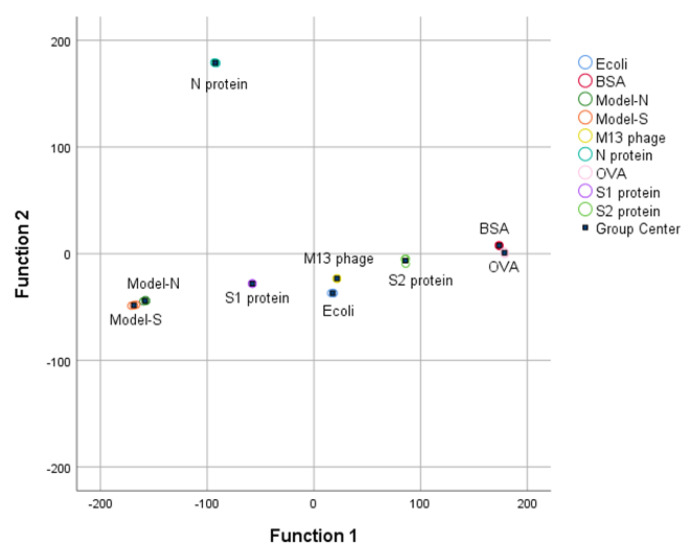
RS Group center plot for the nine samples after LDA analysis.

With the processing of PCA, the nine samples containing the two bio−threats were differentiated through a preliminary dimensionality reduction. Then, LDA was employed based on the result processed from PCA. The discriminant functions were derived in Table 6. Also, it could be seen from the group center diagram that the nine samples were identified and distinguished after PCA−LDA was applied (see Figure 5).

Apart from that, based on the results from cross-validation (see Table 7), 66.7% of Model−N can be identified correctly, and 33.3% for false recognition as Model−S. Beyond that, the rest of the samples could be identified and differentiated exactly with 96.3% accuracy for the classification of the whole samples. Therefore, the combination of RS and PCA−LDA has been an effective and satisfactory method for identifying and discriminating virus−like models and other samples.

## 3. Discussion

Both 3DFS and RS can classify and differentiate the bio-threats from the other samples with the accompany of PCA-LDA algorithms. Moreover, the two methods have been proven reliable through cross-validations with an accuracy of 88.9% and 96.3%, respectively. The correction from RS is slightly higher than that from the 3DFS. However, in 3DFS, misrecognition is among N protein, S2 protein, *E. coli* TG1, and OVA. The synthesized Model-N and Model-S could be separated directly. While in the analysis of RS, the two bio-threats could be recognized from the other samples, it is difficult to identify between them. To be specific, according to the cross-validation, there is a 33.3% probability of misidentification to Model-S for a sample of Model-N. It might cause problems when the individual model is studied. Nevertheless, SARS-CoV-2 is investigated from a holistic perspective, Model-N and Model-S are both derivatives of it, and these models are supposed to imitate the actual viruses as bio-threats and regarded as a whole. Also, there were slight differences among the repeated measurement in 3DFS and RS for an individual sample. However, the overall identification and classification were not affected. The results for both spectra were useful.

## 4. Materials and Methods

### 4.1. Materials

Phagemid vector pHB was from the Academy of Military Medical Sciences. *Sfi*I, *Not*I enzymes, and M13KO7 helper phages were bought from New England Biolabs (Ipswich, MA, USA). ELISA coating buffer and TMB substrate solution were bought from Solarbio. Rabbit anti-SARS-CoV-2 S protein polyclonal antibody was bought from Sino Biological (No. 40592-T62-100) (Beijing, China). Rabbit anti-M13 pAb-HRP was bought from Antaizhiyuan Technology Co., Ltd. (Beijing, China). Syringe Filter Units (0.45 µm) were bought from Merck Millipore (Burlington, MA, USA). Broths and culture media were prepared by ourselves. The automatic microplate reader (SPARK) was bought from TECAN. The molecular interaction analyzer (ForteBio Octet K2, Fremont, CA, USA) was bought from SARTORIUS. FLS1000 Fluorescence Spectrophotometer was from Edinburgh Instruments Ltd (Livingston, UK). Moreover, DXR3 Raman spectrometer was from ThermoFisher Inc (Waltham, MA, USA).

### 4.2. Methods

#### 4.2.1. Acquisition of the Virus-like Models

**Figure 6:**
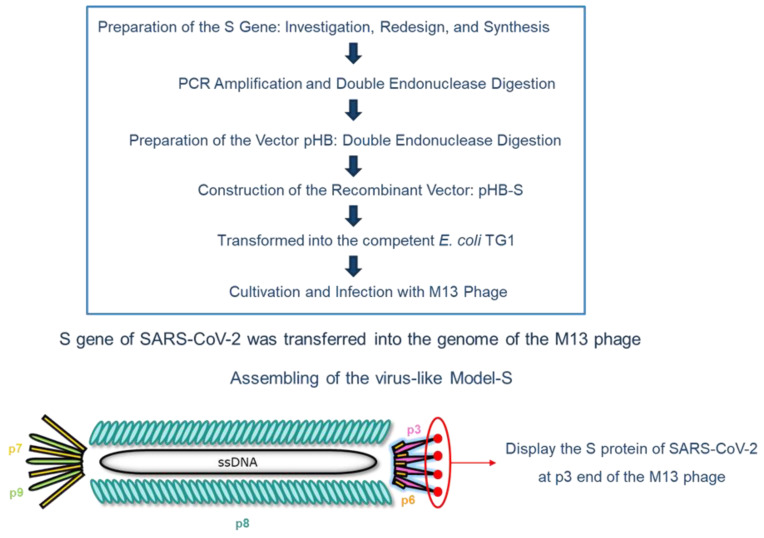
Schematic flow diagram for the preparation of the virus-like Model-S.

In our previous work, the nucleocapsid (N) protein of SARS-CoV-2 was focused on synthesizing the Model-N by phage display. Similarly, in this study, the spike (S) protein was targeted and used to produce the Model-S following the same protocol. The S protein of SARS-CoV-2 has a molecular weight of about 180–200 kDa. Figure 6 illustrates the processing for the synthesis of Model-S.

After searching the gene corresponding to the S protein of SARS-CoV-2 at the National Center for Biotechnology Information (NCBI) website (Gene ID: 43740568), it was modified by adding *Sfi*I and *Not*I restriction digest sites on both sides. After the regular PCR amplification and double endonuclease digestion by *Sfi*I/*Not*I enzyme, the inserted fragment was prepared. The dosage and duration conditions were in accordance with the previous work, as shown in Table 8 (a) and (b) [45].

When the vector pHB was digested by the same enzymes as well, the two parts were constructed and combined with recombinant vector pHB-S produced. The experimental conditions followed the same protocol preparing pHB-N, as presented in Table 9 [45].

Then, it was transformed into the competent *E. coli* TG1. After cultivation, M13 phages were added to infect TG1, and the synthesized Model-S was finally produced after filtrations. The specific cultivation process was described before [45].

#### 4.2.2. Verification of Model-S

(1) Expression: enzyme-linked immunosorbent assay (ELISA)

Sandwich ELISA was applied for the expression verification of the spike protein. Rabbit anti-SARS-CoV-2 S protein polyclonal antibody was used to coat a 96-well plate. Then, the produced Model-S was placed with different diluted ratios after washing and blocking the plates. After that, the Rabbit anti-M13 pAb-HRP was added and aimed to form a sandwich structure. Followed by the reaction with the TMB substrate, OD values at 450 nm were collected and analyzed to decide whether the Model-S could express the spike protein of SARS-CoV-2.

(2) Infection: titer determination

In order to estimate the infection ability of the virus-like model, the plaque assay was used to determine the titer of the produced Model-S since it is the gold standard for titer measurement. The displayed phages Model-S from different dilution ratios were employed to infect *E. coli* TG1. After that, LB solid culture media with kanamycin was applied to provide a space for cultivation. Moreover, the mixtures were cultivated at 37 °C overnight. The plates were collected, and all the plaques were counted to obtain the titer of Model-S the following day.

(3) Affinity: molecular interaction assessment

The molecular interaction instrument ForteBio Octet K2 (SARTORIUS, Göttingen, Germany) was used to assess the combination between the produced Model-S and the corresponding anti-SARS-CoV-2 spike protein antibody (Rabbit pAb). The interaction between these biomolecules could be monitored and collected through the shift of the probe surface reflection interference spectrum following the thin film interferometry technology. The process, including association, dissociation, and regeneration, was performed repeatedly according to the preprogramming. After the measurement, the association constant (ka) and dissociation constant (kd) were obtained. Moreover, the affinity constant could be calculated.

After production and verification, the two virus-like models: Model-N [45] and Model-S, were prepared. Accompanied by these two models (as biothreats), M13 phage, bovine serum albumin (BSA), ovalbumin (OVA), commercial N protein, commercial S1 protein, commercial S2 protein, and *E. coli* TG1 were used as control samples for further study. All selected samples are related to the actual SARS-CoV-2 virus. The produced Model-S and Model-N are the substitutions of SARS-CoV-2 in this research, which could be regarded as the combination of M13 phage (main body) and N/S protein (p3 site). The M13 phage used in the phage display to synthesize the models is a kind of virus that belongs to the virus family, as SARS-CoV-2 does. BSA and OVA are common proteins for scientific research. In addition, the N protein, S1 protein, and S2 protein are the structural proteins of SARS-CoV-2. Furthermore, as common pathogen types, both bacteria and viruses can cause the outbreak of large-scale infectious diseases. Thus, *E. coli* TG1 was also included for identification research since one of the hosts of the M13 phage is *E. coli* TG1, and it was also used to produce the models. Therefore, the nine selected samples are more or less related to the SARS-CoV-2 virus. The higher the relation between the sample and the SARS-CoV-2 virus (or its substitution), the more difficult it might be to distinguish. In general, the nine selected samples, including viruses, proteins, and bacteria, might be representative of the research. The recognition of the Model-S and Model-N among those related samples could provide a possibility for the nondestructive detection and identification of targeted biothreats. Therefore, the nine samples were prepared for spectroscopy analysis.

#### 4.2.3. Acquisition of the Three-Dimensional Fluorescence Spectroscopy (3DFS)

Three-dimensional excitation-emission matrix fluorescence spectroscopy (3DFS) is currently a mature and widely used analysis method. It uses relative fluorescence intensity, excitation wavelength, and emission wavelength as three-dimensional coordinates, which can characterize the relative fluorescence intensity with the change of excitation and emission wavelength. The nine samples were scanned and analyzed in this study by an FLS1000 Fluorescence Spectrophotometer (Edinburgh Instruments Ltd., Livingston, UK). The measurements followed the protocol strictly. After approximately 30 min-preheat, the continuous xenon lamp was stable, and the samples were put into the equipment and ready to start the test. The spectra for every sample were collected at the range of excitation wavelength 240–700 nm and emission wavelength 260–720 nm with 5 nm increments. The experiments were all conducted under room temperature and normal humidity. All the samples were measured repeatedly (3 times), and every measurement lasted for about 20 min. The results were collected and prepared to perform statistical analysis.

#### 4.2.4. Acquisition of the Raman Spectroscopy (RS)

Raman spectrometer analyzes samples qualitatively and quantitatively by detecting Raman scattered light. In this study, a DXR3 Raman spectrometer (ThermoFisher Inc, Waltham, MA, USA) tested and collected the Raman spectrum of the nine samples. The laser wavelength was 784 nm, and the laser power was 10 nW. The measurement was under the help of a 50× objective lens. Moreover, the collection and pre-exposure times were 5 s, with ten times for every sample exposure. The range of Raman shift was selected between 555–3500 cm^−1^. The original data experienced smoothing and normalization for preprocessing to eliminate the influence of the background and noise. Moreover, for every sample, a triplicate spectrum was applied. All the data were collected and prepared for further analysis.

#### 4.2.5. Statistical Analysis and Algorithms Application

Data from 3DFS and RS were submitted to PCA. The entire spectra were used as the original dependent variables, and the nine selected samples were considered independent groups. PCA analysis was implemented to transfer the numerous variables into fewer interrelated indicators, which are the principal component (PC). The scatter plot under PCs coordinates could be obtained. Secondly, the LDA method was applied with the selected PCs instead of the complete spectral information to classify the nine groups with the functions obtained. A comprehensive model with functions for identifying and discriminating the bio-threats was established. Then, cross-validation was employed to evaluate the performance of the constructed system. The data from 3DFS and RS were processed separately, and the results were compared to obtain a better distinguishment. All the data was processed by IBM SPSS Statistics 26.0

## 5. Conclusions

In summary, in order to provide a possible method for the identification of the high pathogenic virus SARS-CoV-2 apart from the traditional biological approaches, spectra analysis and algorithms were employed to realize the aim. First of all, virus-like models were synthesized by phage display technology based on the spike and nucleocapsid protein of SARS-CoV-2. These nonpathogenic models were prepared to replace the actual virus in research, avoiding physical and environmental safety problems. Then, the nine related samples, including the models, protein, virus, and bacteria, were selected for further distinguishment. Three-dimensional fluorescence spectroscopy and Raman spectroscopy were applied to collect their spectral information. With the combination of algorithms PCA-LDA, the nine samples were separated, and the bio-threats (Model-N and Model-S) were identified successfully, with a correction of 88.9% (3DFS) and 96.3% (RS) after cross-validation. The two approaches (3DFS and RS) were useful and reliable for identification. This study provided an idea to diagnose SARS-CoV-2 from the perspective of spectral algorithm analysis. Also, the study proposed a pattern for the research and recognition of the highly pathogenic virus by a non-intrusive method and nondestructive test, which may help to realize monitoring and control of the pandemic at an early stage.

## Figures and Tables

**Table 1:** Titer measurement of Model-S.

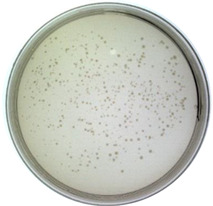	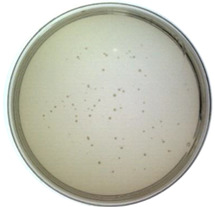	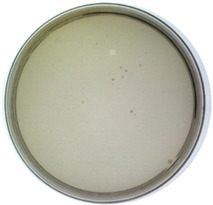
Dilution ratio: 10^5^	Dilution ratio: 10^6^	Dilution ratio: 10^7^
The number of plaques: uncountable	The number of plaques: 102	The number of plaques: 14
Titer: 2.42 × 10^9^ pfu/mL		

**Table 2:** Variance contribution percentage for PCs from 3DFS.

Principle Component/PC	Percentage of Variance/%	Cumulative Percentage/%
1	40.060	40.060
2	26.559	66.619
3	8.357	74.976
4	6.973	81.949
5	4.987	86.936
6	4.000	90.936

Note: Components after the 6th were neglected; they are not shown in this table.

**Table 3:** Functions summarized from LDA and validation for 3DFS.

No.	Function	*p* Value	
1.	f1=3.457×PC1−5.583×PC2+5.544×PC3+8.866×PC4	0.000	Accept
2.	f2=6.649×PC1+1.341×PC2−4.995×PC3+1.011×PC4	0.000	Accept

**Table 4:** Cross-validation for 3DFS.

		Prediction Groups %									
		BSA	OVA	M13 Phage	Model-N	Model-S	N Protein	S1 Protein	S2 Protein	*E. coli* TG1	Total
**Original Groups** **%**	BSA	100.0	0.0	0.0	0.0	0.0	0.0	0.0	0.0	0.0	100.0
	OVA	0.0	100.0	0.0	0.0	0.0	0.0	0.0	0.0	0.0	100.0
	M13 phage	0.0	0.0	100.0	0.0	0.0	0.0	0.0	0.0	0.0	100.0
	Model-N	0.0	0.0	0.0	100.0	0.0	0.0	0.0	0.0	0.0	100.0
	Model-S	0.0	0.0	0.0	0.0	100.0	0.0	0.0	0.0	0.0	100.0
	N protein	0.0	0.0	0.0	0.0	0.0	66.7	0.0	33.3	0.0	100.0
	S1 protein	0.0	0.0	0.0	0.0	0.0	0.0	100.0	0.0	0.0	100.0
	S2 protein	0.0	0.0	0.0	0.0	0.0	33.3	0.0	66.7	0.0	100.0
	*E.coli* TG1	0.0	33.3	0.0	0.0	0.0	0.0	0.0	0.0	66.7	100.0

**Table 5:** Variance contribution percentage for PCs from RS.

Principle Component/PC	Percentage of Variance/%	Cumulative Percentage/%
1	78.575	78.575
2	7.542	86.116
3	4.443	90.559
4	3.805	94.365
5	2.854	97.218
6	1.878	99.096

Note: Data after the 6th PC were omitted.

**Table 6:** Derived functions and validation from LDA analysis for RS.

No.	Function	*p* Value	
1.	f1=2.567×PC1−2.592×PC2−0.080×PC3	0.000	Accept
2.	f2=0.298×PC1−0.500×PC2+0.964×PC3	0.000	Accept

**Table 7:** Cross-validation for RS.

		Prediction Groups %									
		*E. coli* TG1	BSA	Model-N	Model-S	M13 Phage	N Protein	OVA	S1 Protein	S2 Protein	Total
**Original Groups** **%**	*E.coli* TG1	100.0	0.0	0.0	0.0	0.0	0.0	0.0	0.0	0.0	100.0
	BSA	0.0	100.0	0.0	0.0	0.0	0.0	0.0	0.0	0.0	100.0
	Model-N	0.0	0.0	66.7	33.3	0.0	0.0	0.0	0.0	0.0	100.0
	Model-S	0.0	0.0	0.0	100.0	0.0	0.0	0.0	0.0	0.0	100.0
	M13 phage	0.0	0.0	0.0	0.0	100.0	0.0	0.0	0.0	0.0	100.0
	N protein	0.0	0.0	0.0	0.0	0.0	100.0	0.0	0.0	0.0	100.0
	OVA	0.0	0.0	0.0	0.0	0.0	0.0	100.0	0.0	0.0	100.0
	S1 protein	0.0	0.0	0.0	0.0	0.0	0.0	0.0	100.0	0.0	100.0
	S2 protein	0.0	0.0	0.0	0.0	0.0	0.0	0.0	0.0	100.0	100.0

**Table 8:** Conditions for double digestion preparation [45].

*(a) SfiI Digestion System*	
*Target gene (after PCR)*	20 μL
10× buffer1	8 μL
*Sfi*I enzyme	4 μL
Nuclease-free water	43 μL
Total volume	75 μL
Condition: Incubate at 50 °C for 4 h	
*(b) NotI digestion system*	
*System after SfiI digestion*	75 μL
10× buffer2	10 μL
*Not*I enzyme	4 μL
Nuclease-free water	11 μL
Total volume	100 μL
Condition: Incubate at 37 °C for 4 h	

**Table 9:** The combination conditions for producing pHB-S [45].

*Prepared-S-Gene*	*10 μL*
Prepared-pHB	3 μL
10× buffer	5 μL
T4 DNA Ligase	2 μL
Total volume	20 μL
Condition: Incubate at 16 °C overnight	

## Data Availability

Not applicable.

## Supplementary Materials

The following supporting information can be downloaded at: https://www.mdpi.com/article/10.3390/ijms24043209/s1.
