# Supplementary material for: Identify the Virus-like Models for COVID-19 as Bio-Threats: Combining Phage Display, Spectral Detection and Algorithms Analysis
Source: Int J Mol Sci. 2023 Feb 6;24(4):3209. doi: 10.3390/ijms24043209 (PMC9967019; doi:10.3390/ijms24043209)

Supplementary Information

S1. Sequencing validation for the S gene

GGCCCAGCCGGCCATGTTTGTTTTTCTTGTTTTATTGCCACTAGTCTCTAGTCAGT
GTGTTAATCTTACAACCAGAACTCAATTACCCCCTGCATACACTAATTCTTTCACA
CGTGGTGTTTATTACCCTGACAAAGTTTTCAGATCCTCAGTTTTACATTCAACTCA
GGACTTGTTCTTACCTTTCTTTTCCAATGTTACTTGGTTCCATGCTATACATGTCTC
TGGGACCAATGGTACTAAGAGGTTTGATAACCCTGTCCTACCATTTAATGATGGT
GTTTATTTTGCTTCCACTGAGAAGTCTAACATAATAAGAGGCTGGATTTTTGGTAC
TACTTTAGATTCGAAGACCCAGTCCCTACTTATTGTTAATAACGCTACTAATGTTG
TTATTAAAGTCTGTGAATTTCAATTTTGTAATGATCCATTTTTGGGTGTTTATTACC
ACAAAAACAACAAAAGTTGGATGGAAAGTGAGTTCAGAGTTTATTCTAGTGCGA
ATAATTGCACTTTTGAATATGTCTCTCAGCCTTTTCTTATGGACCTTGAAGGAAAA
CAGGGTAATTTCAAAAATCTTAGGGAATTTGTGTTTAAGAATATTGATGGTTATTT
TAAAATATATTCTAAGCACACGCCTATTAATTTAGTGCGTGATCTCCCTCAGGGTT
TTTCGGCTTTAGAACCATTGGTAGATTTGCCAATAGGTATTAACATCACTAGGTTT
CAAACTTTACTTGCTTTACATAGAAGTTATTTGACTCCTGGTGATTCTTCTTCAGG
TTGGACAGCTGGTGCTGCAGCTTATTATGTGGGTTATCTTCAACCTAGGACTTTTC
TATTAAAATATAATGAAAATGGAACCATTACAGATGCTGTAGACTGTGCACTTGA
CCCTCTCTCAGAAACAAAGTGTACGTTGAAATCCTTCACTGTAGAAAAAGGAATC
TATCAAACTTCTAACTTTAGAGTCCAACCAACAGAATCTATTGTTAGATTTCCTAA
TATTACAAACTTGTGCCCTTTTGGTGAAGTTTTTAACGCCACCAGATTTGCATCTG
TTTATGCTTGGAACAGGAAGAGAATCAGCAACTGTGTTGCTGATTATTCTGTCCT
ATATAATTCCGCATCATTTTCCACTTTTAAGTGTTATGGAGTGTCTCCTACTAAAT
TAAATGATCTCTGCTTTACTAATGTCTATGCAGATTCATTTGTAATTAGAGGTGAT
GAAGTCAGACAAATCGCTCCAGGGCAAACTGGAAAGATTGCTGATTATAATTAT
AAATTACCAGATGATTTTACAGGCTGCGTTATAGCTTGGAATTCTAACAATCTTG
ATTCTAAGGTTGGTGGTAATTATAATTACCTGTATAGATTGTTTAGGAAGTCTAAT
CTCAAACCTTTTGAGAGAGATATTTCAACTGAAATCTATCAGGCCGGTAGCACAC
CTTGTAATGGTGTTGAAGGTTTTAATTGTTACTTTCCTTTACAATCATATGGTTTCC
AACCCACTAATGGTGTTGGTTACCAACCATACAGAGTAGTAGTACTTTCTTTTGA
ACTTCTACATGCACCAGCAACTGTTTGTGGACCTAAAAAGTCTACTAATTTGGTT
AAAAACAAATGTGTCAATTTCAACTTCAATGGTTTAACAGGCACAGGTGTTCTTA
CTGAGTCTAACAAAAAGTTTCTGCCTTTCCAACAATTTGGCAGAGACATTGCTGA
CACTACTGATGCTGTCCGTGATCCACAGACACTTGAGATTCTTGACATTACACCA
TGTTCTTTTGGTGGTGTCAGTGTTATAACACCAGGAACAAATACTTCTAACCAGG
TTGCTGTTCTTTATCAGGATGTTAACTGCACAGAAGTCCCTGTTGCTATTCATGCA

GATCAACTTACTCCTACTTGGCGTGTTTATTCTACAGGTTCTAATGTTTTTCAAAC
ACGTGCAGGCTGTTTAATAGGGGCTGAACATGTCAACAACTCATATGAGTGTGAC
ATACCCATTGGTGCAGGTATATGCGCTAGTTATCAGACTCAGACTAATTCTCCTC
GGCGGGCACGTAGTGTAGCTAGTCAATCCATCATTGCCTACACTATGTCACTTGG
TGCAGAAAATTCAGTTGCTTACTCTAATAACTCTATTGCCATACCCACAAATTTTA
CTATTAGTGTTACCACAGAAATTCTACCAGTGTCTATGACCAAGACATCAGTAGA
TTGTACAATGTACATTTGTGGTGATTCAACTGAATGCAGCAATCTTTTGTTGCAAT
ATGGCAGTTTTTGTACACAATTAAACCGTGCTTTAACTGGAATAGCTGTTGAACA
AGACAAAAACACCCAAGAAGTTTTTGCACAAGTCAAACAAATTTACAAAACACC
ACCAATTAAAGATTTTGGTGGTTTTAATTTTTCACAAATATTACCAGATCCATCAA
AACCAAGCAAGAGGTCATTTATTGAAGATCTACTTTTCAACAAAGTGACACTTGC
AGATGCTGGCTTCATCAAACAATATGGTGATTGCCTTGGTGATATTGCTGCTAGA
GACCTCATTTGTGCACAAAAGTTTAACGGCCTTACTGTTTTGCCACCTTTGCTCAC
AGATGAAATGATTGCTCAATACACTTCTGCACTGTTAGCGGGTACAATCACTTCT
GGTTGGACCTTTGGTGCAGGTGCTGCATTACAAATACCATTTGCTATGCAAATGG
CTTATAGGTTTAATGGTATTGGAGTTACACAGAATGTTCTCTATGAGAACCAAAA
ATTGATTGCCAACCAATTTAATAGTGCTATTGGCAAAATTCAAGACTCACTTTCTT
CCACAGCAAGTGCACTTGGAAAACTTCAAGATGTGGTCAACCAAAATGCACAAG
CTTTAAACACGCTTGTTAAACAACTTAGCTCCAATTTTGGTGCAATTTCAAGTGTT
TTAAATGATATCCTTTCACGTCTTGACAAAGTTGAGGCTGAAGTGCAAATTGATA
GGTTGATCACAGGCAGACTTCAAAGTTTGCAGACATATGTGACTCAACAATTAAT
TAGAGCTGCAGAAATCAGAGCTTCTGCTAATCTTGCTGCTACTAAAATGTCAGAG
TGTGTACTTGGACAATCAAAAAGAGTTGATTTTTGTGGAAAGGGCTATCATCTTA
TGTCCTTCCCTCAGTCAGCACCTCATGGTGTAGTCTTCTTGCATGTGACTTATGTC
CCTGCACAAGAAAAGAACTTCACAACTGCTCCTGCCATTTGTCATGATGGAAAAG
CACACTTTCCTCGTGAAGGTGTCTTTGTTTCAAATGGCACACACTGGTTTGTAACA
CAAAGGAATTTTTATGAACCACAAATCATTACTACAGACAACACATTTGTGTCTG
GTAACTGTGATGTTGTAATAGGAATTGTCAACAACACAGTTTATGATCCTTTGCA
ACCTGAATTAGACTCATTCAAGGAGGAGTTAGATAAATATTTTAAGAATCATACA
TCACCAGATGTTGATTTAGGTGACATCTCTGGCATTAATGCTTCAGTTGTAAACAT
TCAAAAAGAAATTGACCGCCTCAATGAGGTTGCCAAGAATTTAAATGAATCTCTC
ATCGATCTCCAAGAACTTGGAAAGTATGAGCAGTATATAAAATGGCCATGGTAC
ATTTGGCTAGGTTTTATAGCTGGCTTGATTGCCATAGTAATGGTGACAATTATGCT
TTGCTGTATGACCAGTTGCTGTAGTTGTCTCAAGGGCTGTTGTTCTTGTGGATCCT
GCTGCAAATTTGATGAAGACGACTCTGAGCCAGTGCTCAAAGGAGTCAAATTAC
ATTACACATAAGCGGCCGC

S2. Affinity analysis of Model-S

The affinity analysis of Model-S was investigated by the molecular interaction instrument ForteBio Octet K2 (SARTORIUS, Germany). Below are the Figure 1 (d) and (e) from the manuscript and the related information was shown below in Table S1.

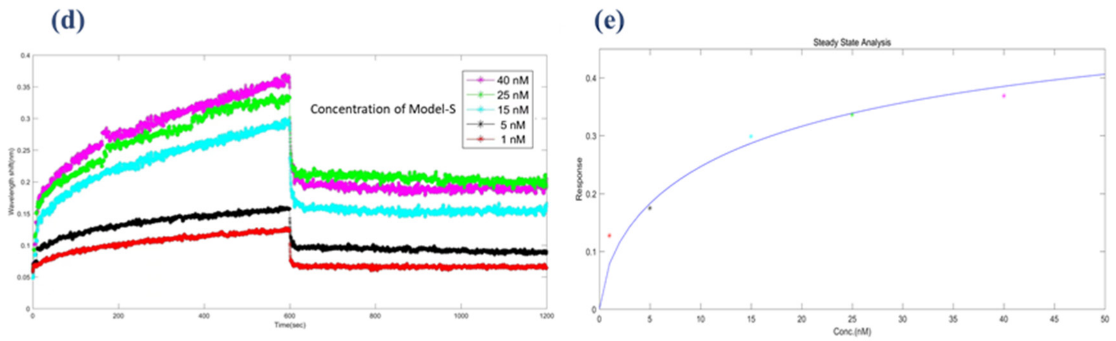

Table S1. Fitting results for the affinity analysis of Model-S

Ka=1.426×10^{5} $M^{-1}s^{-1}$<br>Kd=3.525×10^{-4} s^{-1}	$KD=\frac{Kd}{Ka}=2.473\times10^{-9}$ M	Affinity constant=4.044×10^{8} M^{-1}
Binding model	1:1 Model<br>(Fitting one analyte in solution binding to one binding site on the surface.)	
Model equation	$Response=\frac{R_{max}\times Conc.}{KD+Conc.}$	
Results	$Chi^2/DoF=0.0021$	$R^2=0.9417$

S3. Results from three-dimensional fluorescence spectroscopy for the nine samples.

(1) BSA

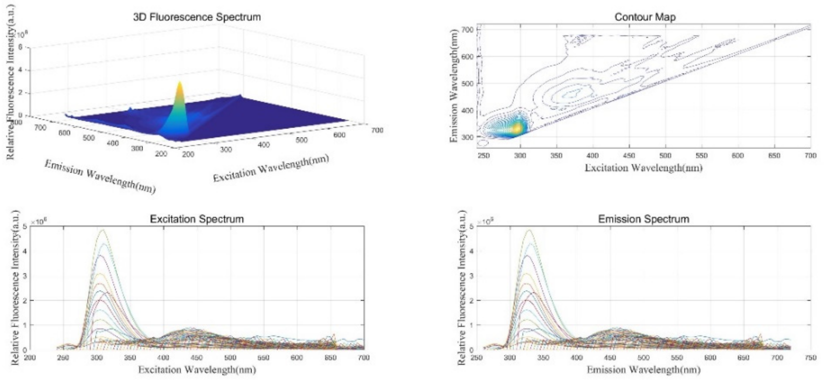

(2) OVA

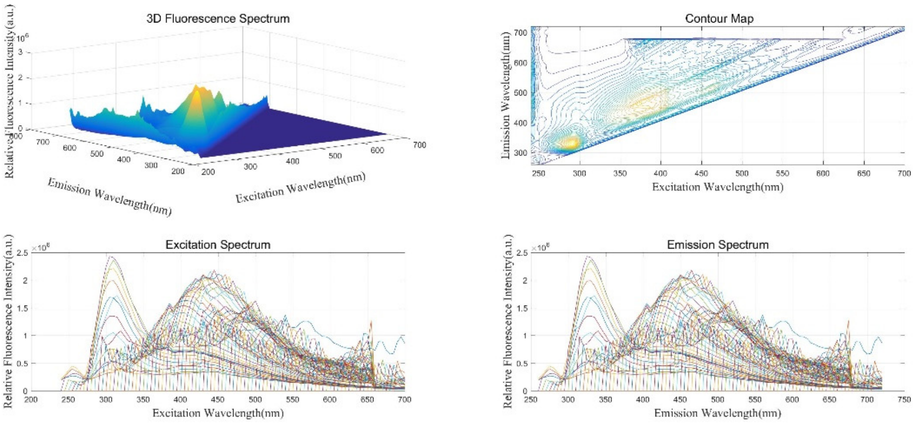

(3) M13 phage

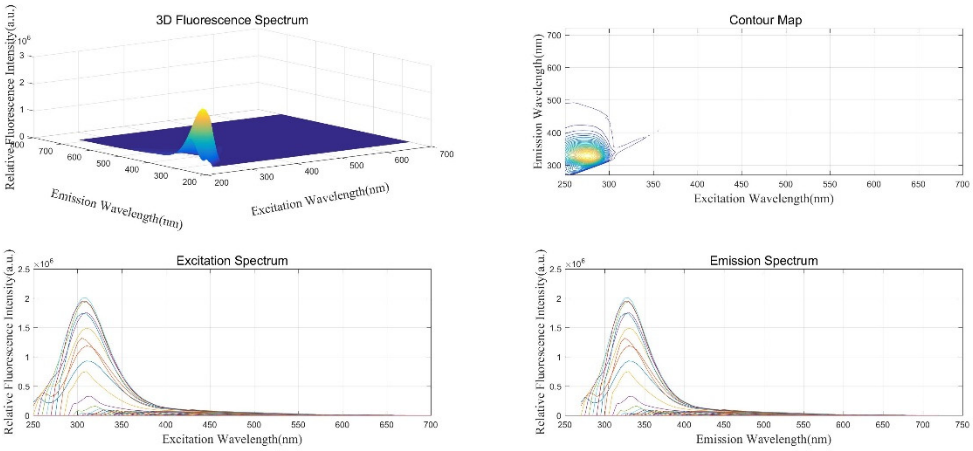

(4) Model-N

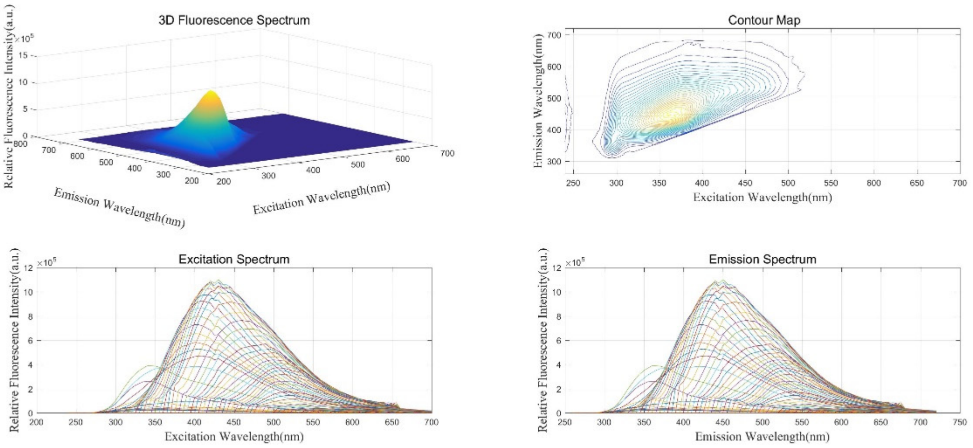

(5) Model-S

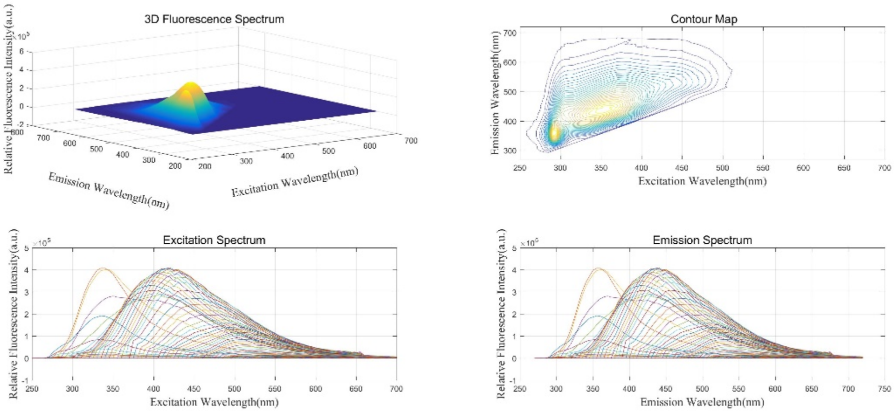

(6) N protein

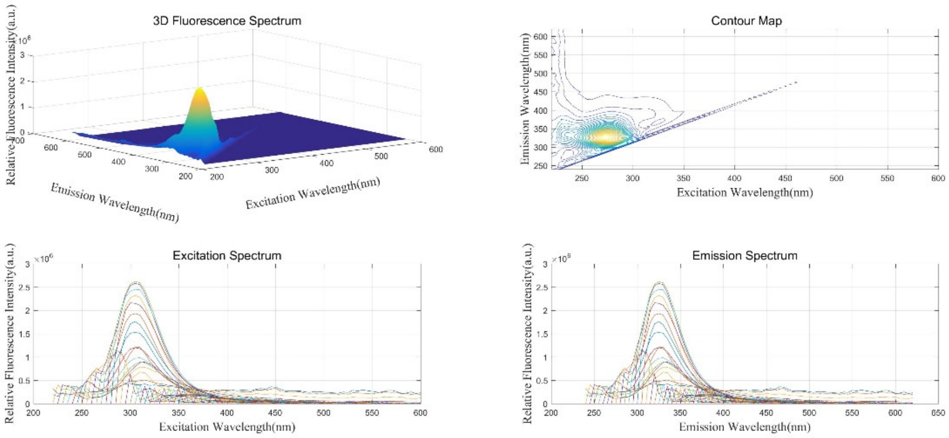

(7) S1 protein

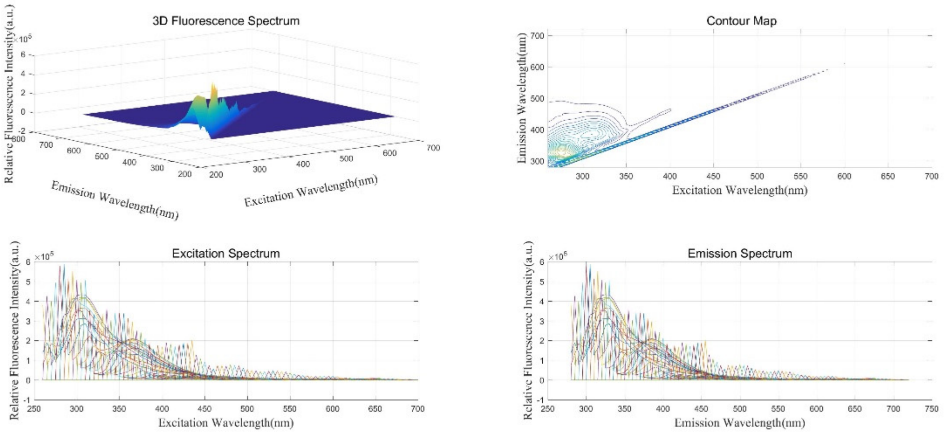

(8) S2 protein

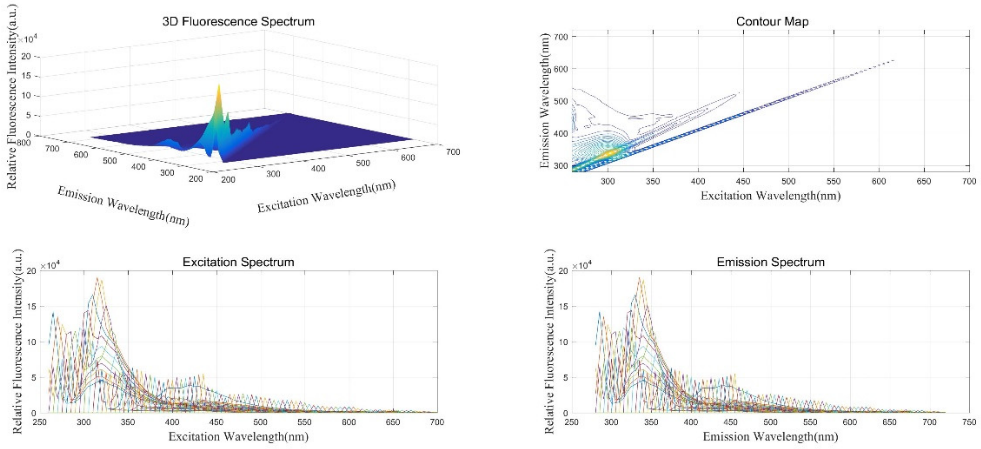

(9) *E.coli* TG1

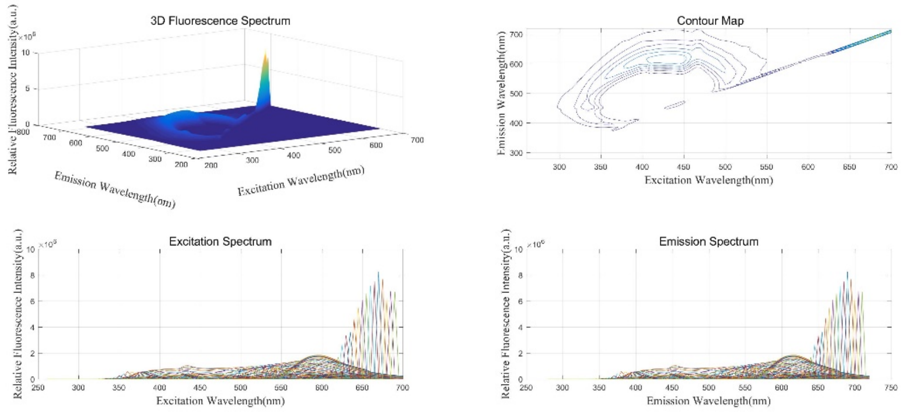

S4. Results from Raman spectroscopy for the nine samples

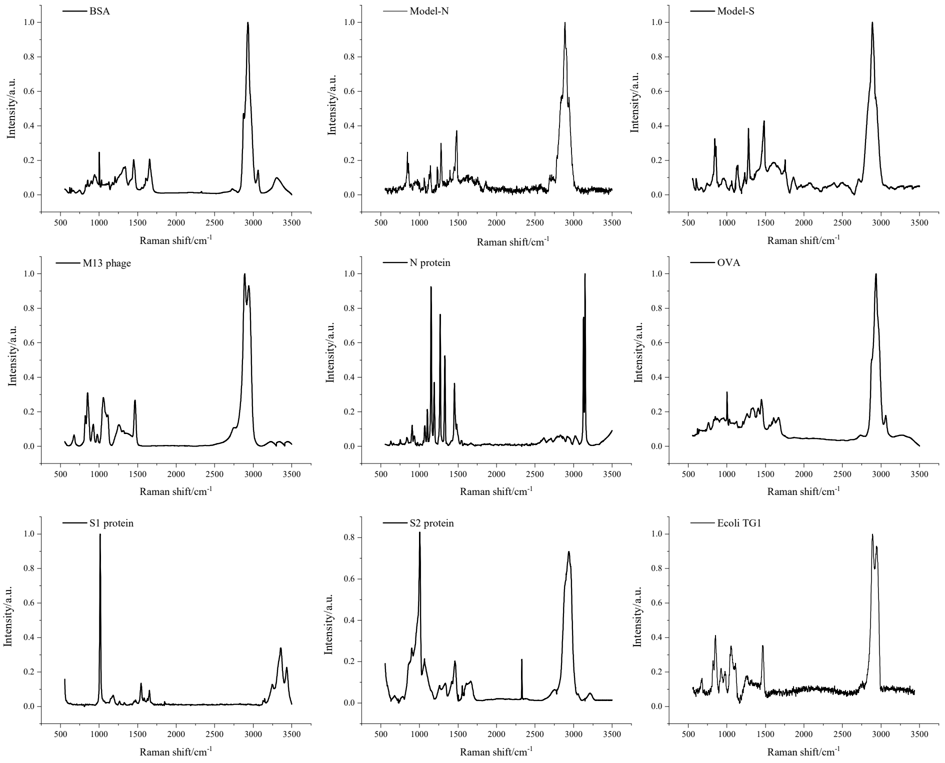

Supplement: Supplementary file 1 [file ijms-24-03209-s001.zip › ijms-2144435-supplementary.pdf]
